# Adaptation of a Culturally Relevant Nutrition and Physical Activity Program for Low-Income, Mexican-Origin Parents With Young Children

**DOI:** 10.5888/pcd12.140591

**Published:** 2015-05-14

**Authors:** Lucia Kaiser, Judith Martinez, Marcel Horowitz, Catherine Lamp, Margaret Johns, Dorina Espinoza, Michele Byrnes, Mayra Muñoz Gomez, Alberto Aguilera, Adela de la Torre

**Affiliations:** Author Affiliations: Judith Martinez, Adela de la Torre, Center for Transnational Health, Chicano Studies, University of California, Davis, California; Marcel Horowitz, University of California Cooperative Extension Yolo County, Woodland, California; Catherine Lamp, University of California Cooperative Extension Tulare County, Tulare, California; Margaret Johns, University of California Cooperative Extension Kern County, Bakersfield, California; Dorina Espinoza, University of California Cooperative Extension Humboldt/Del Norte Counties, Eureka, California; Michele Byrnes, UC CalFresh, University of California, Davis, California; Mayra Muñoz Gomez, Alberto Aguilera, Department of Nutrition, University of California, Davis, California.

## Abstract

Latino children experience higher rates of obesity than do non-Latino white children. Family-centered nutrition interventions can slow the rate of weight gain in this population. *Niños Sanos, Familia Sana* (Healthy Children, Healthy Family) is a 5-year, community-based, participatory research study that targets rural Mexican-origin farmworker families with children aged 2 to 8 years in California’s Central Valley. Adaptation of a culturally relevant obesity prevention program involved qualitative research to tailor key obesity prevention messages, pilot testing and implementation of key messages and activities at family nights, and continual modification to incorporate culturally innovative elements. Of the 238 families enrolled, 53% (125) attended the recommended minimum of 5 (of 10 possible) classes during the first year. A university and community partnership can guide development of a culturally tailored obesity prevention program that is suitable for reaching a high-risk Mexican-origin audience through cooperative extension and other public health programs.

## Introduction

Prevalence of overweight and obesity, defined as body mass index (BMI, weight in kilograms divided by the square of height in meters) at or above the 85th percentile for age and sex, is high among Latino children (1–3). In the United States during 2011 and 2012, the prevalence of overweight and obesity was 38.9% for Latino, 35.2% for non-Latino black, and 28.5% for non-Latino white children aged 2 to 19 years (1). Overweight and obese Latino children have risk factors for nutrition-related chronic diseases. A decline in insulin sensitivity and the prevalence of β cell dysfunction, acanthosis nigricans, high blood pressure, elevated lipid levels, and inflammation may signal an underlying metabolic pathology leading to earlier onset of type 2 diabetes and cardiovascular disease (4–8).

Family-centered interventions can slow weight gain in Latino children (9,10). Successful interventions generally include culturally tailored messages, a focus on parenting skills and nutrition and physical activity behaviors, sufficient exposure, and supportive social environments. A common challenge is sustaining family-level behavior changes through influences at higher spheres of the Social Ecological Model (11), including school and community environments, health care settings, policies, and social norms. Engaging community partners in research is essential to address local barriers to health promotion (12). Each community is unique and the lessons learned can be valuable, although they are not always readily available to other researchers. This article describes the development of a multiyear culturally adapted, nutrition education program for Mexican-origin families using a community engagement approach (13,14). Because this program was designed for Latinos of Mexican descent, we use the terms “Mexican-origin” (US- or Mexican-born) or “Mexican immigrant” (only Mexican-born) to refer to our target audience, recognizing that cultural factors and health may differ among Latino subgroups.

## Needs of the Target Audience

The target audience for the program was Mexican-origin families with young children, residing in 2 rural communities of California’s Central Valley (15–17). These communities lie within one of the nation’s poorest Congressional districts (16). Approximately 89% of the fathers and 87% of the mothers were born in Mexico (17). Sixty-three percent of fathers were seasonally employed farmworkers and 65% of mothers were homemakers. In 2012, 85% of fathers worked within the previous 6 months, and 43% of the families reported food insecurity. According to baseline anthropometric measurements, 51% of the children, aged 2 to 8 years, were overweight or obese.

In 2010, graduate students conducted 3 focus groups with parents in these communities to guide development of a childhood obesity intervention. The parents were not surprised to learn that Mexican immigrant children are heavier than their counterparts living in Mexico. They cited many factors to be related to this phenomenon, including less exercise in the United States, need for both parents to work, poor quality of US foods (“not fresh, too many chemicals”), limited variety of fruits and vegetables available locally, too much fast food, “parents give in too easily” to children’s demands, and poor quality of school food. Many parents identified the transition to school as being a frustrating and worrisome time when their efforts to maintain healthy family meals become more difficult as a result of their children’s rejection of traditional foods.

Another issue that emerged was the high prevalence of food insecurity, which, combined with childhood obesity, underscores the need to address economic and social constraints while drawing on the cultural strengths of this Mexican-origin, farmworker community. Therefore, although the stated goal of the study was to prevent excessive weight gain in young Mexican-origin children, the nutrition education program positioned itself with the broader goal of promoting positive child development — cognitively, socially, and physically — and to maintain close family ties as children grow older.

## Methods

*Niños Sanos, Familia Sana* (NSFS, Healthy Children, Healthy Family) is a 5-year community-based study, aiming to reach 800 children aged 2 to 8 years and their families who reside in California’s Central Valley (15). The institutional review board at University of California, Davis (UC Davis), approved the study protocol. Parents or legal guardians signed informed consent forms in their preferred language (Spanish or English).

By September 2014, the study had completed 1 year of baseline data collection and planning and 2 years (out of 3 planned) of active intervention. In this quasi-experimental study, the 2 matched school districts were randomly assigned to either the intervention or control group. For the 3 years of the intervention, families in the intervention community received a $25 monthly incentive to purchase fruits and vegetables at a local store and attend parent nutrition and physical activity classes (family nights). In the 6 preschools and 2 elementary schools, the intervention children and their classmates received nutrition education offered through the University of California Cooperative Extension (UCCE) and a physical activity program (SPARK) (www.sparkpe.org) taught by the physical education or classroom teacher. Control group families participated in a community-wide art project and family nights on nonnutrition topics, including fostering school success and addressing mental health issues.

The parent education program was delivered in the intervention community. Informed by the social learning theory (18), the NSFS program evolved through 3 stages, with progressively more community leadership at each stage.

### Baseline/planning (July 2011–August 2012)

A nutrition team of UCCE faculty and UC Davis graduate students reviewed the literature on childhood obesity in Latinos and recommendations from the American Academy of Pediatrics (AAP) (19). A graduate student examined existing UCCE curricula to identify useful components and gaps related to childhood obesity prevention. A bilingual graduate student and the project coordinator, a member of the community, conducted 2 focus groups in the intervention community in 2011 to determine how parents interpreted and prioritized key AAP childhood obesity messages.

Considering the children’s young age, parental feedback, and concerns related to child rejection of traditional foods, the team selected 2 primary messages (eat more fruits and vegetables, enjoy home-cooked family meals more often) and 1 secondary message (serve appropriate child-sized portions) for the first year. Anticipating that many children would be in the elementary school system by the second year, the team chose increasing physical activity and reducing screen time and sugar-sweetened beverages as key messages for the second year. Remaining childhood obesity messages related to fast food, sleep, and breakfast were planned for the third year. The nutrition team presented the plan to a local advisory committee, including 2 to 4 community *promotoras* (lay workers) and leaders from the schools, medical clinic, and city office. This committee convened quarterly and gave advice on how to deal with specific issues. For example, *promotoras* suggested that produce from the local community garden be included in the food demonstrations. The committee also recommended ways to present individualized feedback to parents in health reports (eg, emphasize family goals, distribute in small groups).

By July 2012, a local educator with a background in family counseling was hired to lead the family nights in the intervention site. Being strongly committed to an active, healthy lifestyle, the educator became a credible role model for the key messages. She established her credibility by sharing how she had been raised on a less healthy diet in that same community but was able to adopt a healthier diet and lifestyle. From June through August, the educator completed one-on-one nutrition training with a nutrition specialist, a food safety tutorial, and job-shadowing of experienced extension educators.

### Pilot-testing/implementation (September 2012–August 2013)

The parent education classes commenced in September 2012, with a general orientation to the program and use of the monthly $25 fruit and vegetable incentive to be redeemed at a local grocery store. The project coordinator selected a group of 6 participants from among those who were the first to enroll to form a “pilot group.” This group of participants received each lesson before the other families and provided feedback for improvements. Each month (for 10 months each year), a single topic was presented to small groups of parents (9 to 15) on multiple occasions (some evening, some morning classes). Because not all families attended every month, the classes did not build sequentially on the material but rather revisited the key messages throughout the monthly topics. Each class, which lasted about 1 hour, followed a learner-centered format focused on behavior change and consisted of 4 parts:

1. Anchor: to engage participants in the topic, usually by reflection on their current behaviors or memories from childhood.
2. Add: to motivate participants by connecting the key messages to their hopes for their children and by sharing experiences as immigrant families in this community.
3. Apply: to problem-solve, apply skills in hands-on activities, and taste a new recipe with fruits or vegetables.
4. Away: share what was learned and set goals.

**Figure 1:**
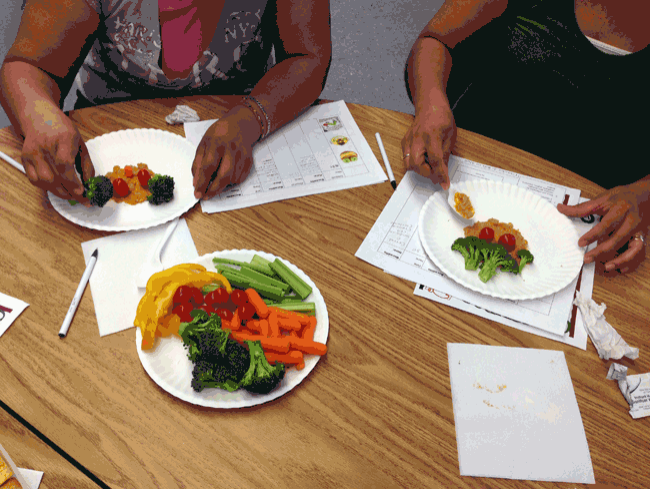
Photograph of a food-preparation activity from a culturally adapted nutrition education program for Mexican-origin families, California’s Central Valley, 2012–2013.

Monthly nutrition team conference calls provided ideas for adapting the content of the lessons on the basis of UCCE experience in delivering nutrition education to low-income audiences from the same cultural background and nearby counties. The educator led the lessons, assisted by a *promotora* who handled the food demonstration and sign-in sheet. Several times per month, a Spanish-speaking nutrition specialist used a checklist to observe the classes. Afterward, the specialist and educator debriefed about coverage and accuracy of key messages, identified activities that evoked the best response, and decided where additional reinforcement was needed. After a single *promotora* with the strongest interest was selected to schedule participants in classes and assist with lessons, debriefing sessions incorporated her input on the effectiveness of the messages, activities, and recipe modifications. In blending perspectives, internal and external to the community, these sessions provided a mix of coaching, content training, and planning and served as a cultural lens to frame obesity prevention messages in meaningful ways. For example, during a discussion on reducing consumption of sugar-sweetened beverages, the educator suggested challenging parents to think about the cultural practice of keeping sodas on hand for visitors and how these beverages become readily available to their children. Stories helped people see how other families find ways to achieve a healthy lifestyle in this rural community. For example, the *promotora* described a typical weekday for her son to show how at least 60 minutes of daily physical activity can be achieved through walking to school, bike riding, and playing ball. Role-playing and skits were other effective and entertaining strategies to show parents how they might talk to their children about food choices (Table, Figure 1).

**Table:** Plan for a Culturally Adapted, Family-Centered Nutrition Education Program, California, 2011–2014

Lesson/Key Message	School’s out! What Will You Feed Your Children? (Summer Example)	Jump up and Move! (Winter Example)
	Eat More Fruits and Vegetables	Increase Physical Activity
Why teach this lesson?	Often both parents are employed in summer agricultural work. Children are left at home in care of older siblings. Time to cook is limited.	Winter season is cold and rainy. Family income is reduced due to seasonal unemployment. Many parents lack awareness of how play can help develop motor skills.
How lesson reinforces earlier lessons	Previous lesson introduces reading food labels.	Previous lesson helps parents identify progression of motor skill development from 2 to 5 years.
Objectives	List healthy foods to buy for snacks. Identify quick, easy recipes to prepare for children’s lunches.	Identify how much physical activity is recommended. Learn physical activities that support motor development and how to increase physical activity at home.
Anchor: ice breaker	Ask: Think back to last summer, what foods did you keep at home for your children to eat while you were working?	Begin with circle game. Engage families to act out different actions (eg, pick a pear from the tree, swim away from a shark). Ask: Which motor skill is used in each action?
Add: key messages	Children tend gain more weight over the summer, especially if already overweight. Why does this happen? What are some easy family suppers and snacks to make this summer?	Explain how much physical activity is recommended for young children and how many children in this community are meeting the goal. Explain benefits of structured and unstructured active play. Ask: What games did you play while growing up in Mexico?
Apply: interactive, hands-on activity	In groups, participants read food labels of several typical snack food items available in local store and sort into 2 bags: healthy choices or unhealthy choices. Ask: why did you sort the foods this way?	Parents and children explore together different activities using recycled or inexpensive, simple materials: bean bag toss, paper plate paddle ball, bowling with water bottles; milk gallon scoop and ball game. Ask: Which motor skills does each support?
Food demonstration	Strawberry yogurt *licuado* (smoothie) with fresh spinach	Oatmeal-apple crisp dessert
Away: goal setting	Make a shopping list of foods	Try at least 2 motor development activities with your child each week.
Children’s activity	Assistant leads children in following simple recipe instructions to make smoothies.	Assistant leads children in games first while parents talk. Children teach games to parents.
Social Cognitive Theory constructs	Have a credible role model (local educator with healthy lifestyle). Teach expectancies about the link between physical activity and normal child motor development. Correct misconceptions about content of snacks commonly purchased by families. Increase self-efficacy through small steps, hands-on activities. Present reciprocal determinism by teaching children and parents to try the same new foods. Self-regulate behavior through goal setting.	

### A “feed-forward” process (September 2013–August 2014)

By September 2013, baseline data from the study became available, enabling the intervention to provide individualized health reports to parents with their children’s weight status and the extent of the problem in their community. The health report used a traffic light design (green = healthy weight, yellow = overweight [85th–94th percentile], and red = obese [≥95th percentile]) to communicate to parents their child’s weight status and included a goal-setting activity, featuring the key obesity prevention messages of the program. During the class, when parents received their children’s health reports, the problem of childhood obesity was framed as being serious and prevalent in this community but also amenable to change through steps that families and communities can take.

As the skills to plan and deliver the program increased, local staff incorporated more culturally relevant, timely elements, on the basis of participants’ responses and suggestions. For example, during a lesson on establishing routines, parents identified the problem of turning off video games at the end of the day as a key challenge. The following month, the educator added a role-playing activity to address this problem. Food demonstrations featured new items, such as eggplant or brown rice, based on the participants’ requests. The educator moved beyond the classroom to disseminate messages at community events, including a school jog-a-thon and the local trick-or-treat event.

**Figure 2:**
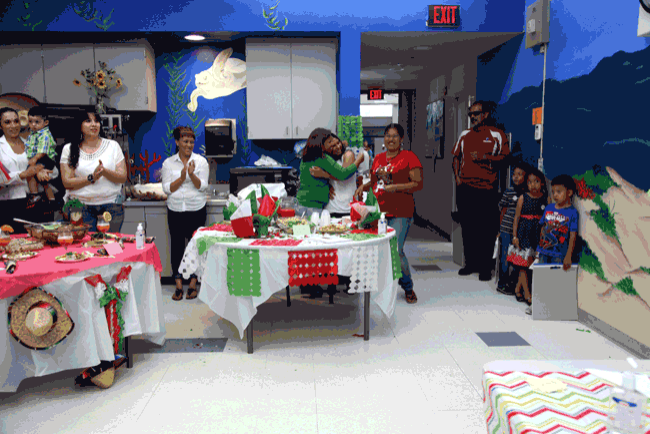
Photograph of a health fair cook-off, an activity from a culturally adapted nutrition education program for Mexican-origin families, California’s Central Valley, 2012–2013.

The culmination of this year was a community health fair event, designed to celebrate healthy living, through booths focused on local nutrition and physical activity resources. The fair included a healthy foods cook-off event for participants and their families. This event was important to help families apply what they had learned, work together in supportive groups, and celebrate healthy traditional foods (Figure 2). The cook-off also presented an opportunity for the school district’s new food service director to build a bridge between the school and the community by sharing school kitchen space for the event, showcasing changes he made in the school lunch program, and serving as a judge in the event.

## Participant Attendance and Feedback

Of the 238 families enrolled during the first year of the intervention, 53% (125) attended a recommended minimum of 5 (out of 10) family nights. In the second year, parent attendance at 5 or more classes dropped to 40% (105) among 263 families enrolled. This decline was most likely because of the lifting of a requirement to attend a minimum of 5 classes each year. Regardless of family night attendance, intervention families continued to receive the vouchers, school-based nutrition education, and physical activity components.

In December 2013, two focus groups were conducted among Spanish-speaking mothers (n = 15) with children who were overweight or obese. Trained in focus methods (20), a bilingual graduate student asked parents about their reactions to the children’s health report cards, probing for any behavior changes. In June 2014, two more focus groups (1 in English and 1 in Spanish, n = 15 mothers, overall) were conducted among participants who had attended 4 or more classes that year. The same graduate student moderated these groups, asking about changes in physical activity and barriers. The interview guides are available from the authors on request.

During the focus groups conducted in December 2013, participants of overweight or obese children reported having attempted to change their family’s diet. They described trying to reduce their children’s consumption of unhealthy foods such as chips, candy, and soda. Many had also introduced new fruits such as kiwis. These parents reported using techniques learned in classes to increase fruit and vegetable consumption, such as arranging food on a plate to grab the child’s attention (Figure 1). However, they noted that outside influences (other family members, food vendors) often made it difficult to maintain dietary changes.

During the focus groups conducted in June 2014, several participants commented that they had used the physical activity games taught in class at home with their children. Participants reported success in decreasing screen time by playing outside with their children, reading books, helping with homework, playing board games as a family, taking computers or televisions out of the bedrooms, and turning the television off after 1 or 2 hours. Some community barriers that prevented participants from an active lifestyle included a lack of youth programs (ie, dance, swim lessons, boxing, and karate), no buildings available in parks to escape bad weather, unsafe walking and biking trails, uneven distribution of parks in the community, and a lack of exercise facilities for adults.

## Conclusion and Implications

University and community partnerships can guide development of culturally tailored obesity prevention programs that are suitable for use in high-risk, Mexican-origin audiences through community programs. Focus group feedback highlighted an immediate need to reach other household members and for environmental improvements at the school and community levels. With these improvements, a curriculum for parents, focused on physical activity and nutrition, can contribute to childhood obesity prevention.
